# Tricarbonyl­chlorido(η^5^-cyclo­penta­dien­yl)molybdenum(II)

**DOI:** 10.1107/S1600536812008471

**Published:** 2012-03-03

**Authors:** Aloice O. Ogweno, Martin O. Onani

**Affiliations:** a University of the Western Cape, Private Bag X17, Bellville 7535, South Africa

## Abstract

The structure of the title compound, [Mo(C_5_H_5_)Cl(CO)_3_], reveals a pseudo-square-pyramidal piano-stool coordination around the Mo^II^ ion, which is surrounded by a cyclo­penta­dienyl ring, three carbonyl groups and a chloride ligand.

## Related literature

For related structures, see: Chaiwasie & Fenn (1968 ▶); Churchill & Bueno (1981 ▶); Albright *et al.* (1978) ▶; Mays & Robb (1968 ▶). For applications of this class of compounds, see: Arzoumanian (1998 ▶); Freund *et al.* (2006 ▶); Karunadasa *et al.* (2010 ▶). For the synthesis, see: Amor *et al.* (2000 ▶); Atwood & Barbour (2003 ▶).
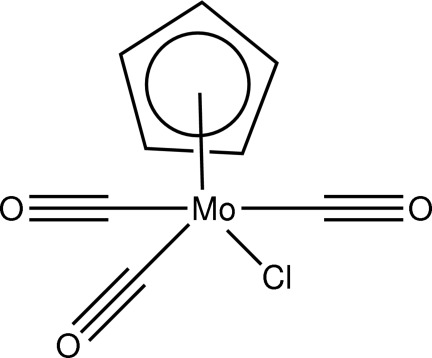


## Experimental

### 

#### Crystal data

[Mo(C_5_H_5_)Cl(CO)_3_]
*M* *_r_* = 280.51
Monoclinic, 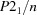

*a* = 6.4958 (6) Å
*b* = 11.7671 (10) Å
*c* = 12.5080 (11) Å
β = 100.064 (2)°
*V* = 941.36 (14) Å^3^
*Z* = 4
Mo *K*α radiation
μ = 1.65 mm^−1^
*T* = 173 K
0.11 × 0.06 × 0.04 mm

#### Data collection

Bruker Kappa DUO APEXII diffractometer
Absorption correction: multi-scan (*SADABS*; Sheldrick, 1997) ▶ *T*_min_ = 0.840, *T*_max_ = 0.937
10440 measured reflections
2355 independent reflections
1953 reflections with *I* > 2σ(*I*)
*R*_int_ = 0.035

#### Refinement

*R*[*F* ^2^ > 2σ(*F* ^2^)] = 0.022
*wR*(*F* ^2^) = 0.046
*S* = 1.01
2355 reflections
118 parameters
H-atom parameters constrained
Δρ_max_ = 0.32 e Å^−3^
Δρ_min_ = −0.30 e Å^−3^

### 

Data collection: *APEX2* (Bruker, 2006 ▶); cell refinement: *SAINT* (Bruker, 2006 ▶); data reduction: *SAINT*; program(s) used to solve structure: *SHELXS97* (Sheldrick, 2008 ▶); program(s) used to refine structure: *SHELXL97* (Sheldrick, 2008) ▶; molecular graphics: *X-SEED* (Barbour, 2001 ▶); software used to prepare material for publication: *SHELXL97* ▶.

## Supplementary Material

Crystal structure: contains datablock(s) I, global. DOI: 10.1107/S1600536812008471/kp2389sup1.cif

Structure factors: contains datablock(s) I. DOI: 10.1107/S1600536812008471/kp2389Isup2.hkl

Additional supplementary materials:  crystallographic information; 3D view; checkCIF report

## Figures and Tables

**Table 1:** Selected bond lengths (Å)

Mo1—C2	1.980 (2)
Mo1—C3	2.008 (2)
Mo1—C1	2.014 (2)
Mo1—Cl1	2.5030 (6)

**Fig. 1.**
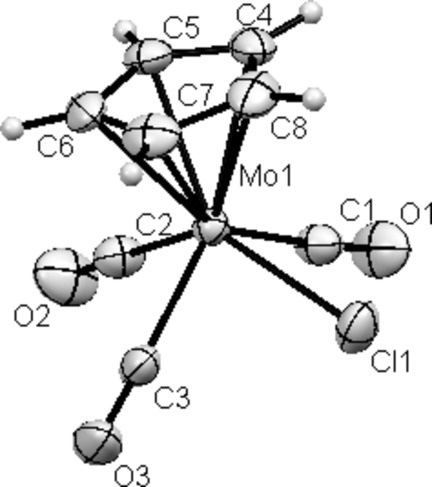
A view of the molecular structure with numbering scheme. Displacement ellipsoids are drawn at the 40% probability level for non-H atoms.

## supplementary crystallographic information

### Comment

The oxo-complexes of transition metals, group 6 are very useful in various catalytic applications. Among the numerous transition metal-oxo compounds that have been used as catalysts, molybdenum is probably the element that stands out as the most investigated for oxygen atom transfer reactions (Arzoumanian 1998). Remarkably, most recently, molybdenum derivative has been used to generate hydrogen from water (Karunadasa *et al.*, 2010). While investigating catalytic epoxidation reactions (Freund *et al.*, 2006), we prepared transition metalcarbonyl complexes containing nitrogen bases, chloro- and cyclopentadienyl(Cp)ligands. This compound could easily be oxidized to the dioxo-molybdenum (IV)complexes without losing the attached ligands. Trying to grow crystals of this complex by slow diffusion in a fridge, the titled compound was obtained insted, probably as a decomposition product. In the titled compound, the ligands display a piano stool arrangement. Notably, the carbonyls and the chloride ligands are spaced by the average angle of 77.49°. The Mo-C2 bond trans to the chloride, Cl1, atom [1.980 (2) Å] is noticeably shorter than the others, Mo–C1, 2.014 (2) and M–C3, 2.008 (2) Å, possibly due to the well-known trans effect. The distance between the Mo atom and the C5, C6 and C7 atoms are observed to be shorter than those between Mo and C4 and C8 because of the electronic repulsion between the electronegative Cl atom and the cyclopentadienyl ring electrons. The molecular structure of the title compound (I) is a new polymorph and differ from the structures reported (Chaiwasie *et al.* 1968; Churchill *et al.* 1981; Albright *et al.* 1978; Mays *et al.*1968). For example, the cell dimensions reported by Chaiwasie *et al.* (1968) is significantly different from our values (see crystal data).

### Experimental

A solution of cyclopentadienyl molybdenum (II) tricarbonyl dimer, [Cp (CO)_3_Mo]_2_, (0.506 g, 1.03 mmol) in THF (10 mL) was added to Na/Hg amalgam in a Schlenck tube with a tap at the bottom. The mixture was stirred until the brick red solution of, [Cp(CO)_3_Mo]_2_ turned pale-green to confirm the formation of [Cp(CO)_3_Mo]^-^ anions. The reduced dimer solution was filtered under nitrogen to another Schlenk tube. An excess CCl_4_ was added and vigorously stired for 30 min. The solvent was removed under vaccum to give a light yellow solid. Yield:0.55 g (61%). The solution of the product in a minimum volume of dichloromethane was allowed to undergo a slow diffusion in an excess of hexane at 277 K for a few days. Block red single crystals suitable for X-ray analysis were obtained.

### Refinement

All non-hydrogen atoms were refined anisotropically. All the hydrogen peaks could be found in the difference electron density maps but were finally placed in idealized positions and refined in riding models with *U*_iso_ assigned 1.2 times those of their parent atoms and the constraint distances of C—H equal to 0.95 Å. The structure was refined to *R* factor of 0.0221.

### Crystal data

**Table:**

[Mo(C_5_H_5_)Cl(CO)_3_]	*F*(000) = 544
*M**_r_* = 280.51	F(000) = 544
Monoclinic, *P*2_1_/*n*	*D*_x_ = 1.979 Mg m^−^^3^
Hall symbol: -P 2yn	Mo *K*α radiation, λ = 0.71073 Å
*a* = 6.4958 (6) Å	Cell parameters from 10440 reflections
*b* = 11.7671 (10) Å	θ = 3.3–28.4°
*c* = 12.5080 (11) Å	µ = 1.65 mm^−^^1^
β = 100.064 (2)°	*T* = 173 K
*V* = 941.36 (14) Å^3^	Block, red
*Z* = 4	0.11 × 0.06 × 0.04 mm

### Data collection

**Table:**

Bruker Kappa DUO APEXII diffractometer	2355 independent reflections
Radiation source: fine-focus sealed tube	1953 reflections with *I* > 2σ(*I*)
Graphite monochromator	*R*_int_ = 0.035
0.5° φ 0.5° φ scans and ω scans	θ_max_ = 28.4°, θ_min_ = 3.3°
Absorption correction: multi-scan (*SADABS*; Sheldrick, 1997)	*h* = −8→8
*T*_min_ = 0.840, *T*_max_ = 0.937	*k* = −15→15
10440 measured reflections	*l* = −16→16

### Refinement

**Table:**

Refinement on *F*^2^	Primary atom site location: structure-invariant direct methods
Least-squares matrix: full	Secondary atom site location: difference Fourier map
*R*[*F*^2^ > 2σ(*F*^2^)] = 0.022	Hydrogen site location: inferred from neighbouring sites
*wR*(*F*^2^) = 0.046	H-atom parameters constrained
*S* = 1.01	*w* = 1/[σ^2^(*F*_o_^2^) + (0.017*P*)^2^ + 0.2175*P*] where *P* = (*F*_o_^2^ + 2*F*_c_^2^)/3
2355 reflections	(Δ/σ)_max_ = 0.002
118 parameters	Δρ_max_ = 0.32 e Å^−^^3^
0 restraints	Δρ_min_ = −0.30 e Å^−^^3^

### Special details

**Table:**

Experimental. crystal mounted on a cryoloop
Geometry. All e.s.d.'s (except the e.s.d. in the dihedral angle between two l.s. planes) are estimated using the full covariance matrix. The cell e.s.d.'s are taken into account individually in the estimation of e.s.d.'s in distances, angles and torsion angles; correlations between e.s.d.'s in cell parameters are only used when they are defined by crystal symmetry. An approximate (isotropic) treatment of cell e.s.d.'s is used for estimating e.s.d.'s involving l.s. planes.
Refinement. *Special details*Geometry. All e.s.d.'s (except the e.s.d. in the dihedral angle between two l.s. planes) are estimated using the full covariance matrix. The cell e.s.d.'s are taken into account individually in the estimation of e.s.d.'s in distances, angles and torsion angles; correlations between e.s.d.'s in cell parameters are only used when they are defined by crystal symmetry. An approximate (isotropic) treatment of cell e.s.d.'s is used for estimating e.s.d.'s involving l.s. planes.Refinement. Refinement of *F*^2^ against ALL reflections. The weighted *R*-factor *wR* and goodness of fit *S* are based on *F*^2^, conventional *R*-factors *R* are based on *F*, with *F* set to zero for negative *F*^2^. The threshold expression of *F*^2^ > σ(*F*^2^) is used only for calculating *R*-factors(gt) *etc*. and is not relevant to the choice of reflections for refinement. *R*-factors based on *F*^2^ are statistically about twice as large as those based on *F*, and *R*– factors based on ALL data will be even larger.

### Fractional atomic coordinates and isotropic or equivalent isotropic displacement parameters (Å^2^)

**Table:**

	*x*	*y*	*z*	*U*_iso_*/*U*_eq_	
Mo1	0.09443 (3)	0.787273 (14)	0.244289 (15)	0.02342 (6)	
Cl1	0.41891 (9)	0.66982 (5)	0.25627 (6)	0.04278 (15)	
O1	0.0004 (3)	0.58701 (16)	0.39597 (16)	0.0539 (5)	
O2	−0.1151 (3)	0.91712 (18)	0.41495 (16)	0.0580 (5)	
O3	0.4393 (3)	0.95672 (15)	0.35712 (18)	0.0560 (5)	
C1	0.0387 (4)	0.6605 (2)	0.34363 (19)	0.0355 (5)	
C2	−0.0421 (4)	0.8694 (2)	0.35115 (19)	0.0366 (5)	
C3	0.3158 (4)	0.89401 (19)	0.3179 (2)	0.0344 (5)	
C4	−0.1349 (4)	0.7141 (2)	0.09365 (19)	0.0388 (6)	
H4	−0.1983	0.6414	0.0946	0.047*	
C5	−0.2134 (4)	0.8169 (2)	0.1275 (2)	0.0363 (5)	
H5	−0.3391	0.8258	0.1557	0.044*	
C6	−0.0732 (4)	0.90483 (19)	0.1122 (2)	0.0385 (6)	
H6	−0.0886	0.9832	0.1273	0.046*	
C7	0.0940 (4)	0.8552 (2)	0.07028 (19)	0.0419 (6)	
H7	0.2126	0.8937	0.0531	0.050*	
C8	0.0520 (4)	0.7379 (2)	0.05865 (19)	0.0446 (6)	
H8	0.1380	0.6837	0.0312	0.053*	

### Atomic displacement parameters (Å^2^)

**Table:**

	*U*^11^	*U*^22^	*U*^33^	*U*^12^	*U*^13^	*U*^23^
Mo1	0.02314 (10)	0.02310 (9)	0.02324 (10)	−0.00117 (7)	0.00194 (7)	0.00350 (8)
Cl1	0.0329 (3)	0.0364 (3)	0.0588 (4)	0.0086 (2)	0.0075 (3)	0.0075 (3)
O1	0.0611 (13)	0.0507 (11)	0.0479 (11)	−0.0172 (9)	0.0045 (10)	0.0232 (9)
O2	0.0513 (12)	0.0742 (14)	0.0516 (12)	0.0026 (10)	0.0174 (10)	−0.0223 (11)
O3	0.0424 (11)	0.0369 (10)	0.0785 (15)	−0.0112 (8)	−0.0177 (10)	0.0079 (10)
C1	0.0326 (13)	0.0396 (13)	0.0320 (13)	−0.0052 (10)	−0.0005 (11)	0.0033 (10)
C2	0.0300 (13)	0.0454 (14)	0.0330 (13)	−0.0002 (11)	0.0018 (11)	−0.0007 (12)
C3	0.0275 (12)	0.0299 (12)	0.0426 (14)	0.0008 (9)	−0.0028 (11)	0.0081 (11)
C4	0.0473 (15)	0.0336 (12)	0.0294 (12)	−0.0057 (11)	−0.0105 (11)	0.0013 (11)
C5	0.0310 (13)	0.0451 (14)	0.0286 (12)	0.0008 (10)	−0.0066 (11)	0.0023 (11)
C6	0.0492 (15)	0.0291 (12)	0.0312 (13)	0.0024 (10)	−0.0096 (12)	0.0081 (10)
C7	0.0450 (15)	0.0535 (15)	0.0261 (12)	−0.0093 (12)	0.0031 (11)	0.0133 (12)
C8	0.0574 (18)	0.0514 (16)	0.0226 (12)	0.0123 (13)	0.0008 (12)	−0.0036 (11)

### Geometric parameters (Å, º)

**Table:**

Mo1—C2	1.980 (2)	O3—C3	1.136 (3)
Mo1—C3	2.008 (2)	C4—C8	1.390 (4)
Mo1—C1	2.014 (2)	C4—C5	1.407 (3)
Mo1—C6	2.280 (2)	C4—H4	0.9500
Mo1—C5	2.288 (2)	C5—C6	1.413 (3)
Mo1—C7	2.318 (2)	C5—H5	0.9500
Mo1—C4	2.352 (2)	C6—C7	1.412 (4)
Mo1—C8	2.363 (2)	C6—H6	0.9500
Mo1—Cl1	2.5030 (6)	C7—C8	1.410 (4)
O1—C1	1.138 (3)	C7—H7	0.9500
O2—C2	1.145 (3)	C8—H8	0.9500
			
C2—Mo1—C3	75.80 (10)	C8—Mo1—Cl1	82.84 (7)
C2—Mo1—C1	78.15 (10)	O1—C1—Mo1	176.8 (2)
C3—Mo1—C1	111.84 (10)	O2—C2—Mo1	177.9 (2)
C2—Mo1—C6	88.85 (10)	O3—C3—Mo1	177.9 (2)
C3—Mo1—C6	99.54 (9)	C8—C4—C5	107.7 (2)
C1—Mo1—C6	141.41 (9)	C8—C4—Mo1	73.28 (14)
C2—Mo1—C5	84.97 (10)	C5—C4—Mo1	69.89 (13)
C3—Mo1—C5	132.35 (9)	C8—C4—H4	126.2
C1—Mo1—C5	106.00 (9)	C5—C4—H4	126.2
C6—Mo1—C5	36.04 (8)	Mo1—C4—H4	122.4
C2—Mo1—C7	122.57 (10)	C4—C5—C6	108.2 (2)
C3—Mo1—C7	95.63 (9)	C4—C5—Mo1	74.84 (14)
C1—Mo1—C7	149.74 (10)	C6—C5—Mo1	71.69 (14)
C6—Mo1—C7	35.74 (9)	C4—C5—H5	125.9
C5—Mo1—C7	59.35 (9)	C6—C5—H5	125.9
C2—Mo1—C4	115.08 (9)	Mo1—C5—H5	119.4
C3—Mo1—C4	154.06 (9)	C7—C6—C5	107.7 (2)
C1—Mo1—C4	93.79 (9)	C7—C6—Mo1	73.59 (14)
C6—Mo1—C4	59.08 (9)	C5—C6—Mo1	72.27 (13)
C5—Mo1—C4	35.26 (9)	C7—C6—H6	126.2
C7—Mo1—C4	58.49 (9)	C5—C6—H6	126.2
C2—Mo1—C8	142.61 (10)	Mo1—C6—H6	119.8
C3—Mo1—C8	123.76 (10)	C8—C7—C6	107.2 (2)
C1—Mo1—C8	114.93 (10)	C8—C7—Mo1	74.20 (14)
C6—Mo1—C8	58.54 (9)	C6—C7—Mo1	70.67 (13)
C5—Mo1—C8	58.07 (9)	C8—C7—H7	126.4
C7—Mo1—C8	35.04 (9)	C6—C7—H7	126.4
C4—Mo1—C8	34.29 (9)	Mo1—C7—H7	120.6
C2—Mo1—Cl1	134.49 (7)	C4—C8—C7	109.2 (2)
C3—Mo1—Cl1	77.86 (7)	C4—C8—Mo1	72.43 (14)
C1—Mo1—Cl1	78.15 (7)	C7—C8—Mo1	70.76 (13)
C6—Mo1—Cl1	131.96 (7)	C4—C8—H8	125.4
C5—Mo1—Cl1	139.05 (7)	C7—C8—H8	125.4
C7—Mo1—Cl1	96.29 (7)	Mo1—C8—H8	123.0
C4—Mo1—Cl1	104.76 (6)		
			
C2—Mo1—C1—O1	111 (4)	C4—C5—C6—C7	−0.9 (3)
C3—Mo1—C1—O1	−180 (100)	Mo1—C5—C6—C7	65.60 (17)
C6—Mo1—C1—O1	38 (4)	C4—C5—C6—Mo1	−66.49 (17)
C5—Mo1—C1—O1	30 (4)	C2—Mo1—C6—C7	161.76 (16)
C7—Mo1—C1—O1	−26 (4)	C3—Mo1—C6—C7	86.37 (16)
C4—Mo1—C1—O1	−4 (4)	C1—Mo1—C6—C7	−129.00 (18)
C8—Mo1—C1—O1	−32 (4)	C5—Mo1—C6—C7	−115.2 (2)
Cl1—Mo1—C1—O1	−108 (4)	C4—Mo1—C6—C7	−77.83 (16)
C3—Mo1—C2—O2	−31 (6)	C8—Mo1—C6—C7	−37.53 (15)
C1—Mo1—C2—O2	86 (6)	Cl1—Mo1—C6—C7	4.16 (18)
C6—Mo1—C2—O2	−131 (6)	C2—Mo1—C6—C5	−83.02 (16)
C5—Mo1—C2—O2	−167 (6)	C3—Mo1—C6—C5	−158.41 (15)
C7—Mo1—C2—O2	−119 (6)	C1—Mo1—C6—C5	−13.8 (2)
C4—Mo1—C2—O2	174 (6)	C7—Mo1—C6—C5	115.2 (2)
C8—Mo1—C2—O2	−159 (6)	C4—Mo1—C6—C5	37.39 (14)
Cl1—Mo1—C2—O2	26 (6)	C8—Mo1—C6—C5	77.69 (16)
C2—Mo1—C3—O3	−94 (6)	Cl1—Mo1—C6—C5	119.38 (14)
C1—Mo1—C3—O3	−165 (6)	C5—C6—C7—C8	1.0 (3)
C6—Mo1—C3—O3	−7 (6)	Mo1—C6—C7—C8	65.74 (17)
C5—Mo1—C3—O3	−24 (6)	C5—C6—C7—Mo1	−64.72 (17)
C7—Mo1—C3—O3	28 (6)	C2—Mo1—C7—C8	−136.98 (16)
C4—Mo1—C3—O3	25 (7)	C3—Mo1—C7—C8	146.30 (16)
C8—Mo1—C3—O3	51 (6)	C1—Mo1—C7—C8	−9.3 (3)
Cl1—Mo1—C3—O3	124 (6)	C6—Mo1—C7—C8	−115.2 (2)
C2—Mo1—C4—C8	150.55 (15)	C5—Mo1—C7—C8	−76.95 (17)
C3—Mo1—C4—C8	40.5 (3)	C4—Mo1—C7—C8	−35.54 (15)
C1—Mo1—C4—C8	−130.76 (16)	Cl1—Mo1—C7—C8	67.94 (15)
C6—Mo1—C4—C8	78.38 (16)	C2—Mo1—C7—C6	−21.80 (19)
C5—Mo1—C4—C8	116.6 (2)	C3—Mo1—C7—C6	−98.53 (16)
C7—Mo1—C4—C8	36.33 (15)	C1—Mo1—C7—C6	105.9 (2)
Cl1—Mo1—C4—C8	−52.03 (15)	C5—Mo1—C7—C6	38.23 (14)
C2—Mo1—C4—C5	33.94 (18)	C4—Mo1—C7—C6	79.64 (16)
C3—Mo1—C4—C5	−76.1 (3)	C8—Mo1—C7—C6	115.2 (2)
C1—Mo1—C4—C5	112.63 (15)	Cl1—Mo1—C7—C6	−176.89 (14)
C6—Mo1—C4—C5	−38.23 (14)	C5—C4—C8—C7	0.2 (3)
C7—Mo1—C4—C5	−80.28 (16)	Mo1—C4—C8—C7	−61.57 (17)
C8—Mo1—C4—C5	−116.6 (2)	C5—C4—C8—Mo1	61.80 (16)
Cl1—Mo1—C4—C5	−168.64 (13)	C6—C7—C8—C4	−0.8 (3)
C8—C4—C5—C6	0.4 (3)	Mo1—C7—C8—C4	62.62 (18)
Mo1—C4—C5—C6	64.41 (17)	C6—C7—C8—Mo1	−63.39 (17)
C8—C4—C5—Mo1	−64.00 (17)	C2—Mo1—C8—C4	−47.2 (2)
C2—Mo1—C5—C4	−149.49 (16)	C3—Mo1—C8—C4	−160.02 (14)
C3—Mo1—C5—C4	144.94 (15)	C1—Mo1—C8—C4	56.46 (17)
C1—Mo1—C5—C4	−73.36 (16)	C6—Mo1—C8—C4	−80.10 (16)
C6—Mo1—C5—C4	115.5 (2)	C5—Mo1—C8—C4	−37.46 (14)
C7—Mo1—C5—C4	77.63 (16)	C7—Mo1—C8—C4	−118.4 (2)
C8—Mo1—C5—C4	36.40 (14)	Cl1—Mo1—C8—C4	129.80 (14)
Cl1—Mo1—C5—C4	16.90 (19)	C2—Mo1—C8—C7	71.2 (2)
C2—Mo1—C5—C6	94.97 (16)	C3—Mo1—C8—C7	−41.62 (19)
C3—Mo1—C5—C6	29.4 (2)	C1—Mo1—C8—C7	174.86 (15)
C1—Mo1—C5—C6	171.11 (15)	C6—Mo1—C8—C7	38.30 (15)
C7—Mo1—C5—C6	−37.90 (15)	C5—Mo1—C8—C7	80.93 (17)
C4—Mo1—C5—C6	−115.5 (2)	C4—Mo1—C8—C7	118.4 (2)
C8—Mo1—C5—C6	−79.13 (16)	Cl1—Mo1—C8—C7	−111.81 (16)
Cl1—Mo1—C5—C6	−98.64 (15)		
